# Cryptotanshinone enhances wound healing in type 2 diabetes with modulatory effects on inflammation, angiogenesis and extracellular matrix remodelling

**DOI:** 10.1080/13880209.2020.1803369

**Published:** 2020-09-01

**Authors:** Min Song, Lu Chen, Lusha Zhang, Chunxiao Li, Joel Wake Coffie, Zhirui Fang, Liyuan Zhang, Shaoxia Wang, Xiumei Gao, Hong Wang

**Affiliations:** a Tianjin State Key Laboratory of Modern Chinese Medicine, Tianjin, China; b Key Laboratory of Pharmacology of Traditional Chinese Medical Formulae, Ministry of Education, Tianjin University of Traditional Chinese Medicine, Tianjin, China; c Tianjin Key Laboratory of Chinese Medicine Pharmacology, Tianjin University of Traditional Chinese Medicine, Tianjin, China; d School of Integrative Medicine, Tianjin University of Traditional Chinese Medicine, Tianjin, China; e Institute of Traditional Chinese Medicine, Tianjin University of Traditional Chinese Medicine, Tianjin, China

**Keywords:** Diabetic wounds, re-epithelialization, CXCL1, CXCL2, VEGF, fibroblast transformation

## Abstract

**Context:**

Cryptotanshinone (CT) is a diterpene quinone compound from *Salvia miltiorrhiza* Bge. Labiatae has been widely used in cardio-cerebral vascular diseases, which could be potentially effective in treating diabetic wounds.

**Objective:**

This study evaluates the wound healing activity of CT by employing an excisional wound splinting model in db/db mice.

**Materials and methods:**

Wounds were induced at the dorsum of non-diabetic (db/+) and diabetic (db/db) mice and treated with sodium carboxymethyl cellulose (CMC-Na) or 300 mg/kg/d CT for 16 days. Wound closure was measured every two days. Body weight, fasting blood glucose, re-epithelialization, granulation, leukocyte infiltration, capillary density, collagen deposition and expressions of CXCL1, CXCL2, VEGF, Ang-1, p-eNOS, eNOS, α-SMA, MMP2 and MMP9 were analysed. Expression of VEGF and tube formation was measured *in vitro* with human umbilical vein endothelial cells (HUVECs).

**Results:**

CT significantly accelerated rate of wound closure, as the contraction ratio increased from 68% (non-treated group) to 83% (CT-treated group) at days 16 post-injury. A significant increase was observed in re-epithelialization and granulation tissue formation. Mechanistically, CT suppressed leukocyte infiltration and CXCL1 and CXCL2 expression. CT treatment also increased blood vessel density and expression level of VEGF, Ang-1 and p-eNOS. *In vitro*, CT boosted expression of VEGF and tube formation of endothelial cells. Moreover, extracellular matrix (ECM) remodelling was enhanced by CT via promoting fibroblast transformation and inhibiting MMP2 and MMP9.

**Conclusions:**

Our study provides evidence that CT could be developed as a potential therapeutic agent for the treatment of chronic diabetic wound healing.

## Introduction

Diabetes is a metabolic disease associated with a large number of vascular complications including inability of wounds to heal (Okonkwo and DiPietro 2017). The prevalence of diabetes is widespread and has been growing in epidemic proportions. Currently, the disease affects 422 million adults worldwide according to the report of the World Health Organization (WHO) (Zubair and Ahmad 2019), but is expected to reach 693 million by 2045 (Cho et al. 2018). Approximately, 1% of diabetic patients per year require lower-limb amputations, which is a major cause of non-traumatic amputation (Rice et al. 2014). Normal wound repair is a series of coordination of intricate and orderly biological and molecular events, consisting of several overlapping stages: inflammation, new tissue formation, re-epithelialization, matrix deposition and remodelling (Qi et al. 2015; Feng et al. 2019). However, diabetic pathological circumstances lead to excessive inflammatory response and dysfunction of endotheliocytes (Liang et al. 2014; Abd El-Khalik et al. 2020). Concomitantly, processes of new tissue formation and re-epithelialization are disrupted, successful tissue repair fail to proceed and wound healing is delayed (Chin et al. 2019).

Previous studies found that molecules inhibiting excessive inflammation and promoting tissue formation such as substance P, heme oxygenase-1, vascular endothelial growth factors (VEGFs) and fibroblast growth factor (FGF), contribute to improved wound healing (Leal et al. 2015; Chen et al. 2016; Zubair and Ahmad 2019). Unfortunately, the best available treatment for chronic wounds only results in about a 50% healing rate often with temporary effect. What is worse, most treatments of diabetic skin ulcers are growth factors, which are costly (Buchberger et al. 2011; Zubair and Ahmad 2019). Therefore, more effective and specific drugs are urgently needed for the prevention and treatment of diabetic skin ulcers.

Danshen (*Salvia miltiorrhiza* Bge. [Labiatae]), a traditional herbal medicine, has been widely used in treating cardiovascular, cerebrovascular and neurodegenerative diseases for centuries (Zhou et al. 2005; Wang et al. 2012; Chen and Chen 2017). Cryptotanshinone (CT) is an active component purified from the root of Danshen (Han et al. 2019). Preclinical studies show that CT has anti-inflammatory, antioxidative, antibacterial and antitumor effects (Jiang et al. 2017; Nagappan et al. 2019). Previous studies have shown that CT accelerates wound closure and inhibits excessive deposition of extracellular matrix (ECM) components (Li et al. 2016). Meanwhile, CT can stimulate glucose uptake and exert anti-obesity and anti-diabetes effects both *in vivo* and *in vitro* (Kim et al. 2007). However, whether CT can accelerate chronic wound healing caused by diabetes has not been demonstrated. In this study, we are devoted to investigate CT’s impact on diabetes induced chronic wound healing and the underlying mechanism. The research may contribute to the potential application of CT in the clinical therapy of diabetic wound healing.

## Materials and methods

### Reagents

CT (purity ≥ 98%) (DY0011) was obtained from Chengdu Desite Biotech Co., Ltd. (Chengdu, China). RIPA buffer (R0010) was purchased from Solarbio (Beijing, China). Avertin (T48402) was purchased from Sigma-Aldrich (St. Louis, MO). Protease inhibitor (0469132001), phosphatase inhibitor (0490837001), phenylmethylsulfonyl fluoride (PMSF, P0100), Transcriptor First Strand cDNA Synthesis Kit (4897030001) and FastStart Universal SYBR Green Master (4913914001) were bought from Roche (Mannheim, Germany). Trizol reagent (15596-026) was purchased from Life Technology (Waltham, MA). β-Actin (4970S) antibody was obtained from Cell Signaling Technology (Boston, MA). Matrix metallopeptidase 2 (MMP2, WL01579a) antibody was bought from Wanleibio (Shenyang, China). Other antibodies including VEGF (ab46154), CD45 (ab10558), Angiopoietin-1 (Ang-1, ab8451), matrix metallopeptidase 9 (MMP9, ab38898), α-smooth muscle actin (α-SMA, ab5694) and Goat Anti-Rabbit IgG H&L (Alexa Fluor^®^ 594) (ab150080) were provided by Abcam (Cambridge, UK). *Griffonia simplicifolia* Baill. (Caesalpiniaceae) lectin (AL-1103), anti-soybean agglutinin (AS-2014) and dylight^®^594 anti-goat IgG (H + L) (A11012) were obtained from Vector Laboratories (Burlingame, CA). C-X-C motif chemokine ligand 1 (CXCL1, abs131503) and rabbit anti-goat IgG-FITC (abs20006) were bought from absin (Shanghai, China). C-X-C motif chemokine ligand 2 (CXCL2, AF-452-SP) was from Novus (Plymouth, MN). VEGF ELISA kit (DVE00) was obtained from R&D Systems (Minneapolis, MN).

### Animals

Adult male diabetic mice (Strain: BKS.Cg-Dock7^m+^/^+^Lepr^db/Nju^) aged 8 weeks with blood glucose (32.14 ± 2.24 mM) and body weight (51.35 ± 4.08 g) and their control littermates with blood glucose (11 ± 3.67 mM) and body weight (26.48 ± 0.44 g) (db/db and db/+, respectively), were obtained from the Model Animal Research Center at Nanjing University. The protocols for *in vivo* study with mice were approved by the Animal Ethics Committee of Tianjin University of Traditional Chinese Medicine (TUTCM20170311) and performed in accordance with the approved guidelines on the use of laboratory animals. Mice were housed in pathogen-free conditions at the Animal Center of Institute of Biomedical Engineering, Chinese Academy of Medical Sciences (Tianjin, China). They were maintained at controlled temperature (22–25 °C) and relative humidity (50–60%) on a 12 h light/dark cycle with free access to food and water and 3–5 mice per cage.

### Surgical procedures and treatment

All the mice were anaesthetized by intraperitoneal injection of Avertin (16.5 mL/kg). The excisional wound splinting model was generated according to the method described previously (Wang et al. 2013). After hair removal from the dorsal surface under anaesthesia, 6-mm full-thickness excision skin wounds were created on the midline of mice. A donut-shaped silicone splint was fixed around the wound and sewed up with 5-0 suture line. After treating gentamicin on the wounds, the wounds were covered with Tegaderm sterile transparent dressing to provide a waterproof, sterile barrier to external contaminants including liquids, bacteria and viruses and maintain a moist environment for wound healing. It was changed every two days. Then, 60 mice with skin wounds were divided into three groups: (1) control group (*n* = 20). Non-diabetic mice (db/+) were orally administered with sodium carboxymethyl cellulose (CMC-Na) solution; (2) vehicle group (*n* = 20). The db/db mice received CMC-Na solution orally; (3) CT group (*n* = 20). The diabetic mice were administrated with 300 mg/kg/d CT by gavage. CT was dissolved in 0.1% CMC-Na solution and given daily starting on day 0. CT and CMC-Na solution were given for 16 consecutive days.

### Wound closure analysis

Wound closure was measured by tracing the wound area every two days using a camera. Wound closure was quantified by Image J software (Bethesda, MD) and wound healing was expressed as the percentage of the original wound area that had healed, calculated as [1 – (wound area day *x*/wound area day 0)]×100%.

### Histological assessments

Wound skin tissues were fixed with 4% paraformaldehyde, embedded in paraffin and cut into sections (5 μm). They were incubated overnight at 60 °C and dehydrated with graded ethanol series for haematoxylin/eosin (H&E) staining and Masson’s trichrome staining. For immunohistochemical staining, the sections were covered with 3% H_2_O_2_ for 15 min at room temperature and antigen was retrieved by heat mediation for 15 min in a citrate buffer. After blocking with 5% bovine serum albumin (BSA) in Tris-buffered saline for 30 min at 37 °C, the sections were incubated with primary antibodies against CD45 (1:100 in PBS) at 4 °C overnight. Then, the slides were covered with biocatalytic secondary antibody (1:200 in PBS) for 30 min at 37 °C and streptavidin–horseradish peroxidase for another 15 min. Staining was visualized after incubation with a DAB–H_2_O_2_ solution. The slides were then counterstained with haematoxylin for 1 min, dehydrated with ethanol, and sealed in resinene for microscopic observation.

For immunofluorescent labelling, mice were intravenously injected with 50 µL *Griffonia simplicifolia* lectin I (diluted with 1 mL HBS buffer) 30 min before sacrifices. Sections were incubated with preheated antigen retrieval buffer for 15 min. Blocking was done by 5% bovine serum for 1 h at 37 °C. Primary antibody to anti-soybean agglutinin (1:100 in HBS) was incubated with the sections at 4 °C overnight. Secondary antibody DyLight^®^594 Anti-goat IgG (H + L) (1:200 in HBS) was then incubated with the sections for 30 min at 37 °C. Finally, the sections were viewed and photographed using a Nikon TI-U fluorescence microscope (Minato City, Japan).

### Immunoblotting

Immunoblotting analyses were performed to detect the phospho-endothelial nitric-oxide synthase (p-eNOS), eNOS, VEGF, Ang-1, MMP2 and MMP9 protein expressions at day 16 after surgery. The skin tissues at the edge of the wounds were homogenized in RIPA lysis buffer (50 mM Tris–HCl, pH 7.4, 150 mM NaCl, 1% Triton X-100, 0.1% SDS, 1% sodium deoxycholate, 1 mM sodium vanadate, 1 mM PMSF, 10 μg/mL aprotinin, 10 μg/mL leupeptin and 10 mM sodium fluoride) with a cocktail of protease and phosphatase inhibitors using tissue homogenizers on ice. After centrifugation at 12,000 rpm for 15 min at 4 °C, protein samples were obtained from tissue and subjected to quantification using bicinchoninic acid (BCA) assay. Protein (40 μg) was separated on 10% sodium dodecylsulfate polyacrylamide gel electrophoresis (SDS-PAGE) and transferred onto polyvinylidene fluoride (PVDF) membranes. Blocked with 5% BSA or 5% milk in Tris-buffered saline Tween-20 (TBST) (0.1% Tween-20 in TBS) for 3 h, the membranes were incubated with respective primary antibodies at 4 °C overnight and the membranes were incubated with horseradish peroxidase-conjugated secondary antibody (1:10,000) for 1 h at room temperature. The blots were developed by enhanced chemiluminescence detection regents. Gray intensity of protein bands was quantified with Image J (Bethesda, MD) and normalized to that of β-actin in each sample.

### Quantitative real-time reverse transcription polymerase chain reaction (qRT-PCR)

qRT-PCR analyses were performed via standard techniques. Briefly, total RNA was extracted with Trizol Reagent and reverse transcribed with a Transcriptor First Strand cDNA Synthesis Kit. Amplification was performed on a QuantStudio6 Q6 real-time PCR systems (Applied Biosystems, Foster City, CA, USA) by using FastStart Universal SYBR Green Master (Rox). Expression was calculated via the comparative-threshold cycle method and normalized to GAPDH or β-actin mRNA levels. The following primers were used: Mouse CXCL1 (forward: TCGTCTTTCATATTGTATGGTCAAC; reverse: CGAGACGAGACCAGGAGAAAC), Mouse CXCL2 (forward: TGAACAAAGGCAAGGCTAACTG; reverse: GAGGCACATCAGGTACGATCC), Mouse GAPDH (forward: CTTCACCACCATGGAGAAGGC; reverse: GGCATGGACTGTGGTCATGAG), Human VEGF (forward: AGCCTTGCCTTGCTGCTCTAC; reverse: TGATGATTCTGCCCTCCTCCTT), Human β-actin (forward: AAGATCAAGATCATTGCTCCTCCTG; reverse: AGCTCAGTAACAGTCCGCCT).

### Cell culture and tube formation

Human umbilical vein endothelial cells (HUVECs) were purchased from ATCC (ATCC^®^ PCS-100-010™, Manassas, VA) and maintained in EGM-2 medium (Lonza cc-3202, Allendale, NJ) supplemented with 5% foetal buffer saline (FBS). HUVECs were maintained at 37 °C with 5% CO_2_. The tube formation assay was performed according to manufacturer’s protocols of Corning^®^ Matrigel^®^ Matrix (cat. no. 354234, Corning, Corning, NY). For preparation, matrigel matrix was fully dissolved at 4 °C overnight. The medium was then removed and 50 μL Matrigel Matrix was added to each well of 96-well plates and then incubated at 37 °C for 1 h. Then, cell culture medium containing 2 × 10^4^ HUVECs was seeded on the matrigel with dimethylsulphoxide (DMSO) or 2.5 μM CT in each well. After 18 h, the tube formation of HUVECs was observed and photographed using an inverted phase-contrast microscope in five random fields.

### Enzyme-linked immunosorbent assay (ELISA) analysis

After the cells were treated with CT for 24 h, the supernatants of cell cultures were collected and assayed for VEGF using ELISA kit according to the manufacturer’s protocol.

### Statistical analysis

Statistical analysis was performed using SPSS software (version 16.0, Chicago, IL). The results from three independent experiments were expressed as mean ± SD. Differences between two groups were compared with Student's *t*-test. Values of *p* < 0.05 were considered to be statistically significant.

## Results

### CT accelerated wound healing and promoted granulation tissue formation and re-epithelialization *in vivo*

**Figure 1.**
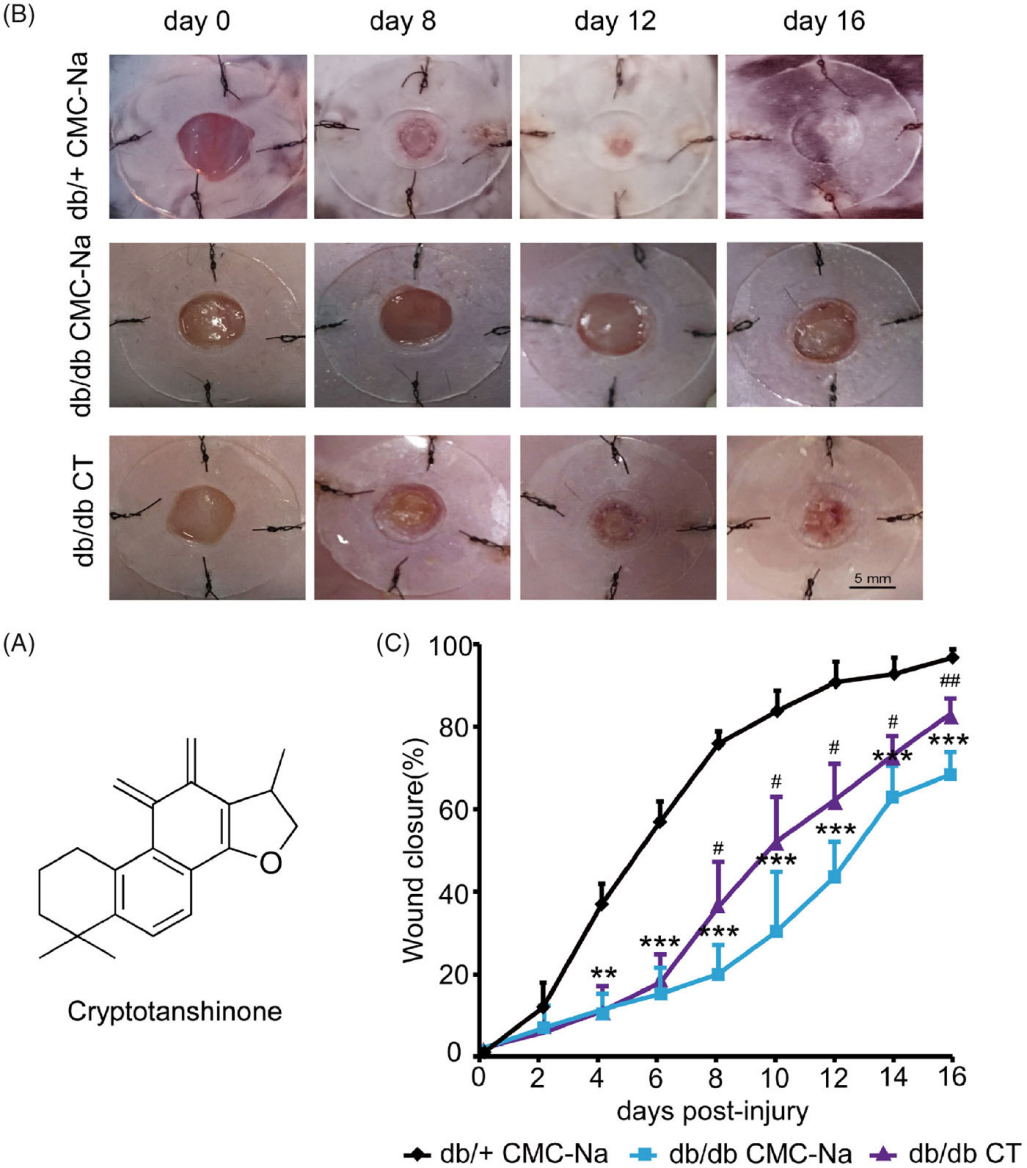
Effect of CT on wound closure in db/db mice. (A) The molecular structure of CT. (B) Representative photographs of wounds on day 0, 8, 12 and 16. Scale bar = 5 mm. (C) The percentage of wound closure area at day 0–16 post-injury. Results were presented as mean ± SD. **p< 0.01 and ***p< 0.001 vs. non-diabetic mice treated with CMC-Na; #*p* < 0.05, ##*p* < 0.01 vs. vehicle mice treated with CMC-Na. *n* = 8.

CT is a diterpene quinone compound extracted from *Salvia miltiorrhiza*. Its molecular structure is presented in Figure 1(A). In order to confirm whether CT has an effect on chronic wound healing induced by diabetes, full-thickness excision wounds were created on the back of 8-week-old BKS.Cg-Dock7m+/+Leprdb/Nju (db/db) mice. These mice are leptin receptor deficient and represent a type II diabetes model characterized with obesity, hyperglycaemia and impaired wound healing. We routinely recorded body weight and fasting blood glucose in diabetic mice. There were no significant differences between CMC-Na group and CT treated group, indicating CT did not affect blood glucose within 16 days. The wound closure was examined every two days. The data reflected that the healing time in the db/+ control group was the shortest. The wound almost healed on day 12. The wound areas of the db/db mice were significantly increased than those in the db/+ mice from day 4 to day 16 (Figure 1(B,C); *p* < 0.05, *p* < 0.01 or *p* < 0.001, respectively). CT treatment improved wound healing rates in comparison with the diabetic CMC-Na-treated group animals from day 8 to day 16 (Figure 1(B,C); *p* < 0.05, *p* < 0.01, respectively). On day 8, granulation tissue (GT) growth was obvious in CT-treated group.

**Figure 2.**
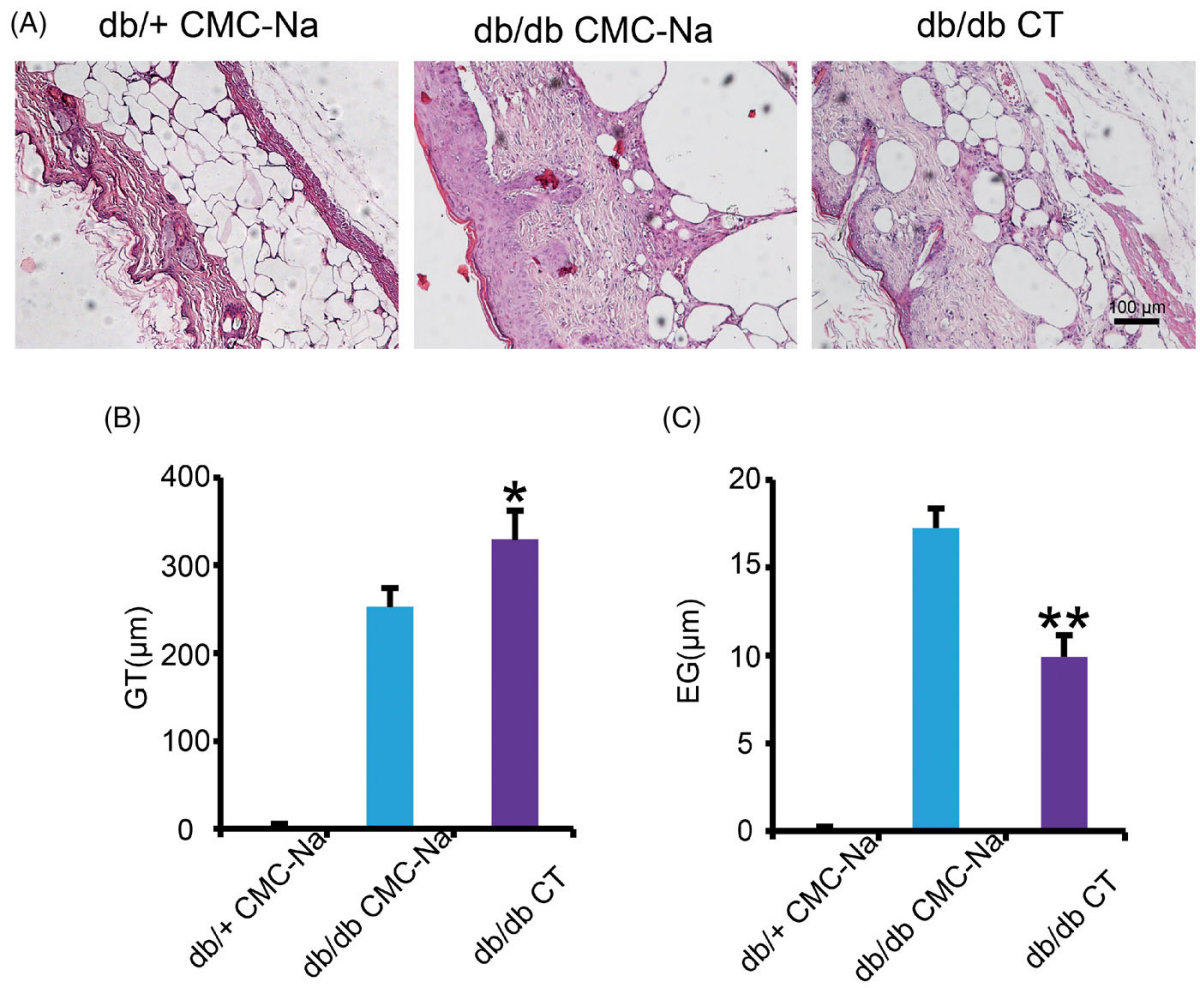
Effect of CT on granulation tissue formation and re-epithelialization in db/db mice. (A) H&E staining of diabetic mice wounds at day 16 post-injury. Scale bar = 100 μm. (B, C) Quantitative calculation of the GT and EG at day 16 post-injury. GT: granulation tissue; EG: epithelial gap. Results were presented as mean ± SD. *p < 0.05, **p < 0.01 vs. vehicle mice treated with CMC-Na, *n* = 6.

Based on the anatomical structure of skin, granulation is a new connective tissue which occurs from the base of the wound and contains microscopic blood vessels. The epithelial gap represents distance between the leading edge of migrating keratinocytes. It is the process of covering open wounds with new epithelial surfaces to create a barrier between the wound and the external environment. They are measured in H&E-stained sections of wounds displayed in Figure 2(A). In the CT-treated wounds, GT is thicker and re-epithelialization was faster compared with CMC-Na-treated wounds at day 16 post-wounding. Quantitative calculations of the GT thickness and re-epithelialization of the wounds confirmed that CT treatment significantly enhanced GT thickness and decreased epithelial gap (Figure 2(B,C); *p* < 0.05, *p* < 0.01, respectively). Therefore, in addition to wound contraction, CT treatment significantly promoted GT formation and re-epithelialization in open wounds.

### Effect of CT on excessive inflammatory response

**Figure 3.**
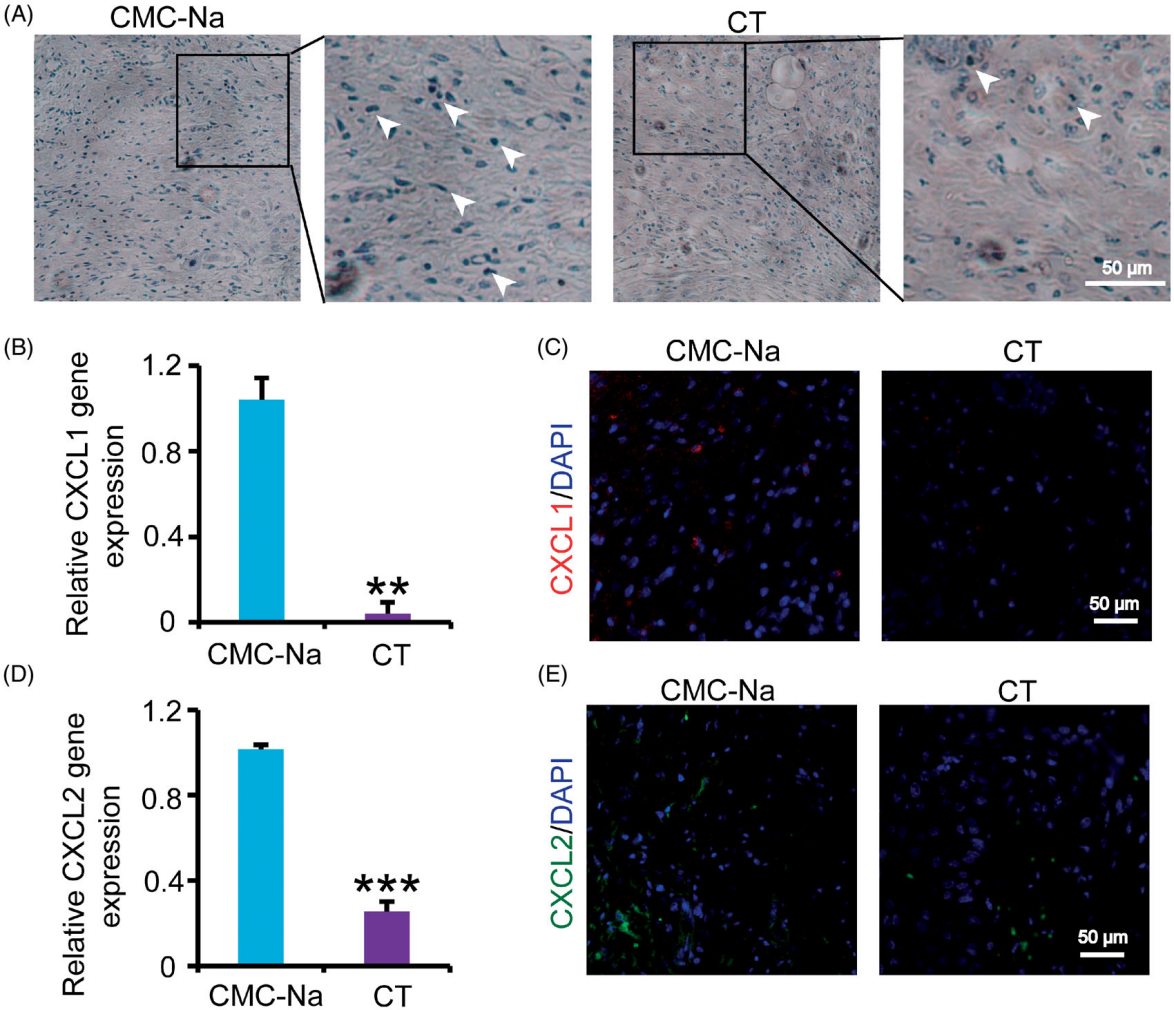
Effect of CT on inflammation in diabetic wounds. (A) Immunostaining of CD45 of diabetic mouse wounds at day 16 post-injury. White arrows indicate the CD45 positive cells. Scale bar = 50 μm. (B) Relative gene expression of CXCL1 in wounds at day 16 post-injury. (C) Immunofluorescence for CXCL1 at day 16 post-injury. Scale bar = 50 μm. (D) Relative gene expression of CXCL2 in wounds at day 16 post-injury. (E) Immunofluorescence for CXCL2 at day 16 post-injury. Scale bar = 50 μm. Results were presented as mean ± SD. **p < 0.01 and ***p < 0.001 vs. vehicle mice treated with CMC-Na, *n* = 3.

Appropriate inflammatory response can promote cell recruitment and tissue regeneration. However, excessive inflammatory response will lead to delayed wound healing in diabetes. To verify whether CT could inhibit the excessive inflammatory response, CD45 staining and relative gene expression were detected by immunohistochemistry and qRT-PCR. The results showed that leukocyte infiltrates were significantly decreased in group receiving CT (Figure 3(A)). CXCL1 and CXCL2 are pro-inflammatory chemokines, which are expressed in inflammatory stage of wound healing. Inhibition of inflammatory cytokines is a main principle for diabetic wounds treatment in the whole process of therapy. CT can significantly reduce the expression of CXCL1 and CXCL2 at day 16 post-injury compared with those in vehicle group (Figure 3(B–E); *p* < 0.01 or *p* < 0.001, respectively), which is compatible with the reduction of the lesion observed in Figure 1, indicating CT may accelerate wound healing through inhibiting inflammation.

### Effect of CT on angiogenesis both *in vivo* and *in vitro*

**Figure 4.**
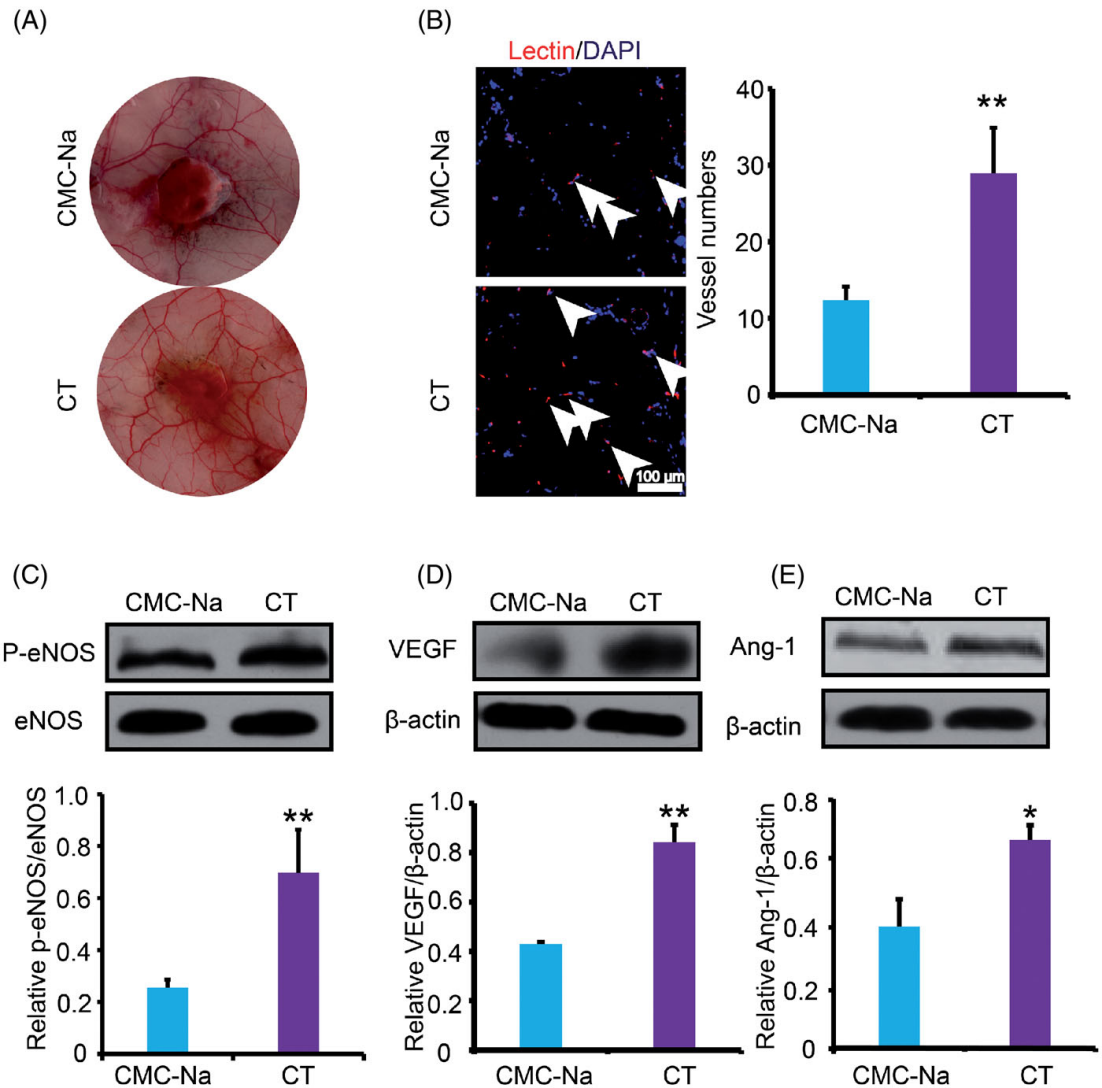
Effect of CT on neovascularization in diabetic wounds. (A) Pictures of blood vessels around the wound. (B) Immunofluorescence for lectin at day 16 post-injury. White arrows indicate the lectin positive cells. Scale bar = 100 μm. (C, D) Western blot of eNOS, P-eNOS, VEGF and Ang-1 and quantitative analysis of P-eNOS/eNOS, VEGF and Ang-1 in the wounds at day 16 post-injury. Results were presented as mean ± SD. *p < 0.05 and **p < 0.01 vs. vehicle mice treated with CMC-Na, *n* = 3.

One reason of impaired wound closure in diabetes is lack of angiogenesis (Zins et al. 2010). Disruption in the neovascularization process consecutively leads to wound healing disturbances. To detect the effect of CT on angiogenesis, the newly formed microvascular networks of a regenerating skin wound were observed at day 16 post-injury. It was found that the microvasculature density around the wounds of CT-treated group was more significant than the vehicle group (Figure 4(A)). Moreover, the histological assessment around the wound was performed. Capillary density was evaluated by identifying lectin^+^ vessels. Remarkably, the overall capillary density in marginal wound regions 16 days post-wounding was significantly higher in the CT-treated mice compared with the CMC-Na-treated animals (*p* < 0.01) (Figure 4(B)). To elucidate the molecular mechanism, western blot analyses were performed to detect the p-eNOS, VEGF and Ang-1 protein expressions at day 16 after surgery. VEGF is an important cytokine involved in creating new blood vessels. eNOS can regulate cell survival, migration, tube formation and NO release, which is essential for the repair of tissue damage. Ang-1 acts as a chemoattractant for endothelial cells while also promoting endothelial cell sprouting and facilitating tissue invasion by nascent blood vessels. As shown in Figure 4(C–E), treatment of CT significantly increased eNOS phosphorylation (*p* < 0.01) and augmented VEGF and Ang-1 protein expression (*p* < 0.01 or *p* < 0.05, respectively). The above results indicated that CT increased neovascularization and the acceleration of improved angiogenesis in CT-treated diabetic wounds may be explained, at least in part, by the activation of pro-angiogenic factors.

**Figure 5.**
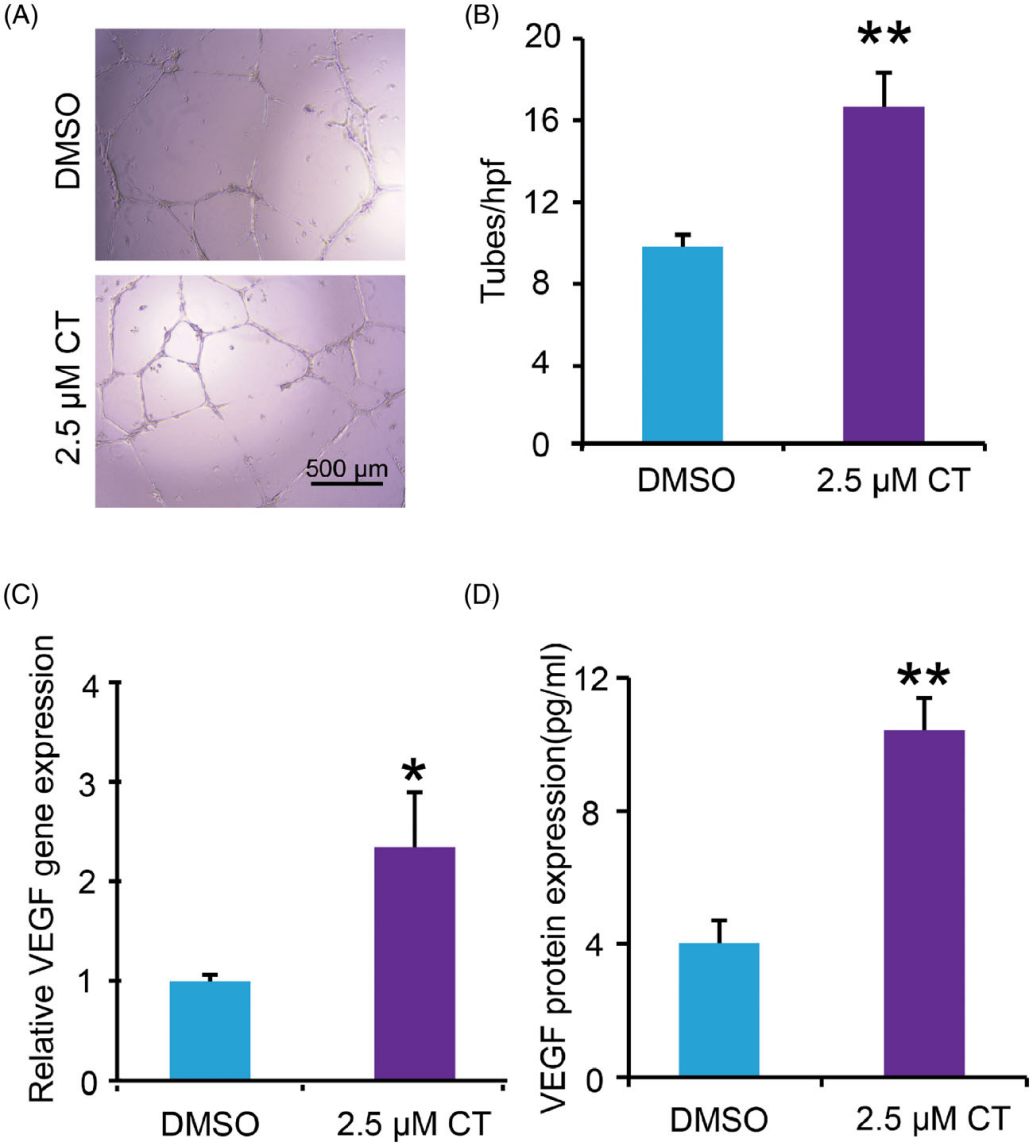
Impact of CT on expression of VEGF and tube formation of HUVECs. (A, B) Tube formation in DMSO and 2.5 μM CT treated groups and quantitative measurement of tube formation. Scale bar = 500 μm. (C, D) VEGF gene and protein expression in DMSO and 2.5 μM CT treated groups. Results were presented as mean ± SD. *p < 0.05 and **p < 0.01 vs. DMSO treated group, *n* = 3.

To further assess endothelial function, the tube-forming activity and VEGF expression were examined with HUVECs. The results showed that CT boosted tube formation (Figure 5(A,B); *p* < 0.01) and promoted VEGF expression and release significantly (Figure 5(C,D); *p* < 0.05, *p* < 0.01, respectively). Taken together, these data confirmed the angiogenic capacity of CT *in vitro*.

### Effect of CT on collagen deposition

**Figure 6.**
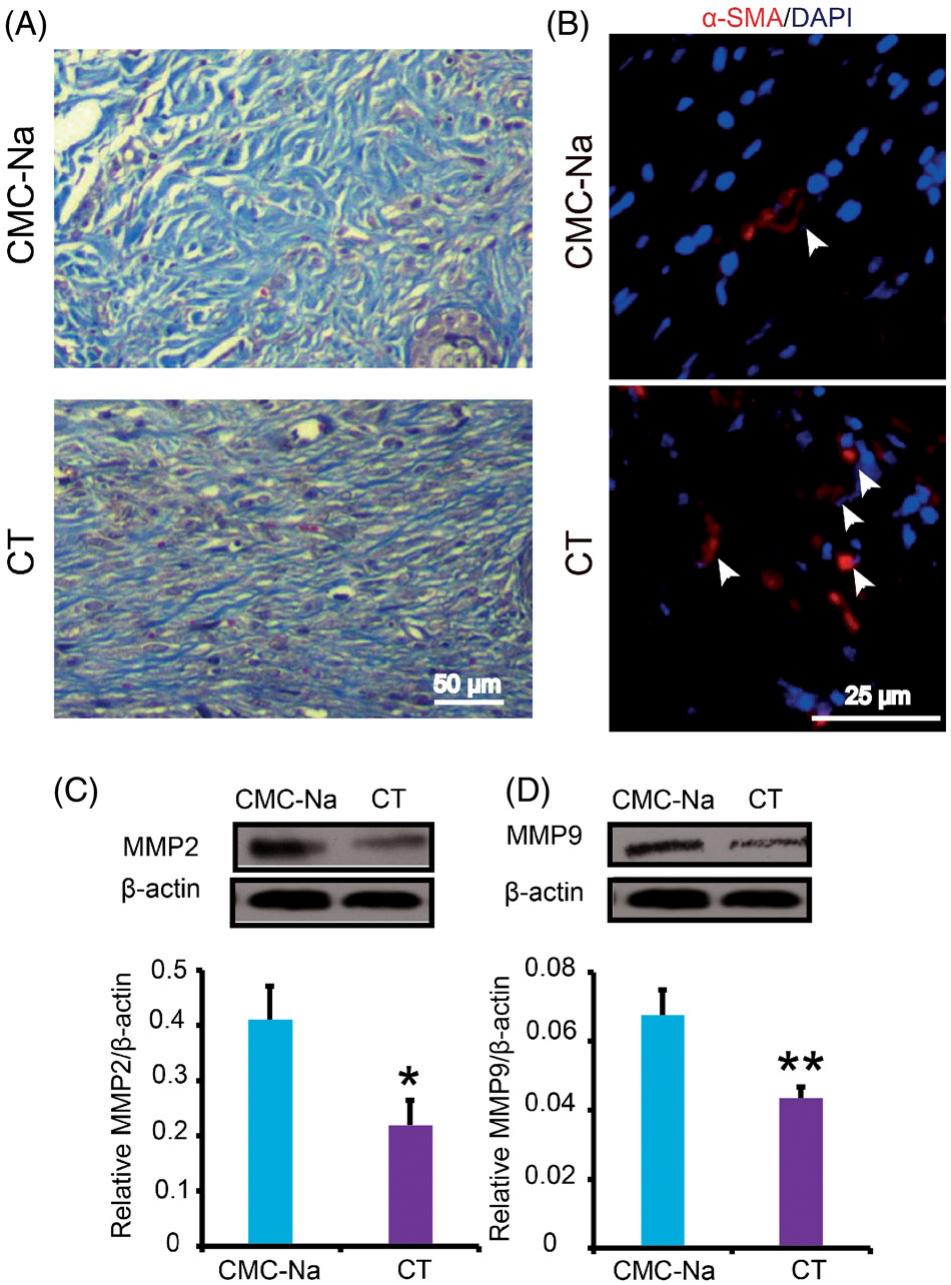
Effect of CT on collagen production in diabetic wounds. (A) Masson’s trichrome staining wounds treated with CMC-Na and CT at day 16 post-injury. CT increased collagen fibres in the wounds compared with CMC-Na. Scale bar = 50 μm. (B) Immunofluorescence for α-SMA at day 16 post-injury. White arrows indicate the α-SMA positive cells. Scale bar = 25 μm. (C, D) Western blot of MMP2 and MMP9 in CMC-Na- or CT-treated wounds and quantitative assay of MMP2 and MMP9 in CMC-Na- or CT-treated wounds at day 16 post-injury. Results were presented as mean ± SD. *p < 0.05 and **p < 0.01 vs. CMC-Na, *n* = 3.

Collagen deposition is a vital function in promoting the formation of ECM and wound healing. In order to explore whether CT can promote collagen deposition, collagen fibres in the wound were observed by Masson staining. The results showed that more collagen fibres were regularly arranged in CT-treated wounds at day 16 post-injury compared with CMC-Na-treated group (Figure 6(A)). In the new tissue formation and maturation, myofibroblasts play a key role by increasing formation of ECM molecules. Meanwhile, they can directly participate in mechanical wound closure due to their contractility. The immunofluorescent results reflected that CT induced dramatical increase in content of α-SMA positive myofibroblasts (Figure 6(B)). MMP-2 and MMP-9 are two active forms of matrix metalloproteinases that are capable of degrading ECM components and are involved in tissue remodelling and restructuring. Both of them prevent the wound from healing and contribute to delayed or impaired wound healing. Our results showed that protein expression level of MMP2 and MMP9 in CT-treated wound was obviously decreased (Figure 6(C,D); *p* < 0.05 or *p* < 0.01, respectively). The above results indicated that ECM was enhanced by CT via promoting fibroblast transformation and inhibiting MMP2 and MMP9.

## Discussion

Natural wound injury initiates acute inflammatory response, mainly involving neutrophils, macrophages, which aggregate to the wound surface, secreting cytokines and growth factors (Hart 2002; Sukeishi et al. 2017; Zubair and Ahmad 2019). With the assistance of cytokines and growth factors, ECM degrades, endothelial cells proliferate, fibroblasts migrate to wound and proliferate (Zhang et al. 2017; Zubair and Ahmad 2019). In addition, accumulated by soluble growth factors, fibroblasts convert into myofibroblasts which synthesize collagen to compose new ECM and facilitate wound closure (Ayuk et al. 2016). Abnormal pathology in diabetes, including excessive inflammation, dysfunction of endotheliocytes and fibroblasts causes obstructed angiogenesis and collagen synthesis, which delay wound healing or give rise to non-healing wound (Lerman et al. 2003). Here, we established an excision wound splinting model, in which the splint was tightly fastened to the skin around the wound, avoiding uniform wound closure owning to skin contraction, therefore, the wound healed through granulation and re-epithelialization, a process similar to that in humans (Wang et al. 2013). Our results revealed that CT accelerated wound closure, including epidermal regeneration, GT formation, neovascularization and ECM remodelling, but did not affect weight and fasting blood glucose in diabetic mice. Mechanistically, CT depressed leukocyte infiltration and expression of chemokine, increased eNOS phosphorylation, VEGF, Ang-1 protein expression, inhibited MMP2 and MMP9 protein expression and enhanced fibroblasts translation resulting in enhancive angiogenesis and collagen deposition in diabetic mice. *In vitro*, low concentration of CT upgraded expression of VEGF and boosted tube formation of HUVECs. Taken together, these observations suggest that CT could be a useful drug for therapeutic chronic wound healing induced by diabetes.

Abundant evidence indicates that disproportionate inflammation which is keenly linked to sustained leukocyte infiltration is a constant trait of diabetes induced wound healing impairment. Thus, inhibition of excessive inflammation can be considered as a therapeutic approach for diabetic complications (Fahey et al. 1991). In our present study, CT receded leukocyte infiltration and restrained gene expression of chemokines CXCL1 and CXCL2 in diabetic wound. The study further proved that CT attenuates excessive inflammation and shortens persistence of inflammation during delayed wound healing induced by diabetes.

Neovascularization has been shown to play a crucial role in wound repair, the formation of new blood vessels provides nutritional basis for newly formed GTs. Insufficient angiogenesis is considered as important causative factor for diabetic wound healing impairment (DiPietro 2016). In high-glucose environment, oxidative stress is activated in endothelial cells by advanced glycation end products (AGEs) and activated oxidative stress can increase reactive oxygen species in blood, and reduce pro-angiogenic factors (Stavrou 2008; Kolluru et al. 2012). eNOS is the major factor for NO synthesis, which is an essential molecule for maintaining the biological functions of endothelial cells, and researchers have found that impaired diabetic wounds showed reduced activation of eNOS (Stallmeyer et al. 2002; Duda et al. 2004). VEGF and Ang-1 are essential proteins for stimulating angiogenesis and vascular maturation in improving course of wound healing and promote endothelial cells migration, proliferation and tube formation. However, in diabetes, the expression of Ang-1 and VEGF are down-regulated. Previous studies have shown that increased VEGF and Ang-1 can accelerate wound healing in diabetic mice (Drela et al. 2012; Losi et al. 2013). In this study, capillary density was increased in CT treated wounds, implying that CT boosted local vessel growth and maturation in the diabetic wounds. CT dramatically increased levels of p-eNOS/eNOS, VEGF and Ang-1 and low concentration CT boosted expression of VEGF and tube formation in the diabetic wounds and in HUVECs, which also illustrated that CT expedites the formation of new blood vessels and is partially responsible for wound healing in db/db mice.

In the middle and late stage of wound healing, some of the fibroblasts in the injured tissue may differentiate into myofibroblasts, which secrete large amounts of collagen fibres, promoting ECM remodelling. Myofibroblasts have bundles of α-SMA which together contribute to the closure of wound (Berry et al. 1998). Degradation of the constituents of the ECM by matrix metalloproteinases (MMPs), leads to wound healing impairment (Caley et al. 2015). In our study, CT treatment increased collagen content in the wound of diabetic mice probably through decrease in protein expression of MMP2 and MMP9. The above results suggested that CT treatment increased collagen content in the wound of diabetic mice probably through decrease in protein expression of MMP2 and MMP9. The above results suggested that CT inhibits the degradation of ECM and accelerates diabetic wound healing.

## Conclusions

We demonstrated that CT accelerated wound healing and re-epithelialization, GT development in diabetic mice by improving angiogenesis and relative protein expression of p-eNOS, VEGF and Ang-1. Moreover, in the process of diabetic wound healing, CT prevented excessive inflammatory response and enhanced ECM remodelling. These results provide evidence for potential clinical application of CT in diabetic wound healing.
